# Congenital brucellosis in a preterm infant: A case report from France and literature review

**DOI:** 10.1016/j.idcr.2026.e02510

**Published:** 2026-02-09

**Authors:** Lisa Ozcan, David Malorey, Lise Martin Perceval, Mona Leroux, Constance Bridonneau, Stéphane Corvec, Valentin Pineau, Cyril Flamant, Jean-Philippe Lavigne, Elise Launay

**Affiliations:** a Limoges University Hospital, Pediatric Care Unit, Limoges, France; b Nantes University, Nantes University Hospital, Pediatric and Pediatric Infectious Diseases Department, Nantes, France; c Nantes University, Nantes University Hospital, Microbiology Department, Nantes, France; d Nantes University, Nantes University Hospital, Infectious and Tropical Diseases Department, Nantes, France; e Nantes University, Nantes University Hospital, Pediatric and Neonatal Intensive Care Unit, Nantes, France; f VBIC, INSERM U1047, Univ Montpellier, Department of Microbiology and Hospital Hygiene, CNR Brucella, Nîmes University Hospital,, Nîmes, France

**Keywords:** Pediatric infectious disease, Brucella, Congenital, Newborn, Zoonosis, Case report

## Abstract

**Introduction:**

Congenital brucellosis is a rare but serious transplacental infection.

**Patients and methods:**

Brucellosis is a bacterial zoonosis, that has become extremely rare in France unlike many countries in the Mediterranean basin, where it remains endemic and is a cause of travel-related morbidity. We have reported the first case of neonatal brucellosis in France since 1990, involving a preterm infant born at 26 weeks with Brucella melitensis chorioamnionitis, due to mother-to-fetal transmission. A PubMed review (2000–2024) identified 13 additional neonatal cases.

**Results:**

The mother had consumed unpasteurized cheese during early pregnancy in Spain. Maternal Blood cultures at delivery were positive for *B. melitensis*. The neonate remained asymptomatic until day 13, when blood culture -negative at birth- became positive. Despite delayed treatment, clinical respone was favorable. Since 2000, most cases have involved *B. melitensis*, presenting with respiratory distress, sepsis, or hepatomegaly. Infants were mainly treated with Rifampicin and TMP-SMX for six weeks, with overall favorable outcomes despite occasional severe or chronic cases.

**Conclusion:**

This rare case highlights the need to consider brucellosis in maternal fever or unexplained neonatal sepsis, with travel and dietary history guiding early diagnosis.

## Highlights

• First documented case of congenital brucellosis in France in the 21st century.
• A typical presentation in a preterm infant with delayed onset of clinical symptoms.
• Consider Brucella in maternal fever or neonatal sepsis with travel or raw dairy exposure.
• PCR allows rapid diagnosis, while blood culture remains the gold standard.
• Empirical neonatal therapy including rifampin is advised in maternal infection.

## Introduction

Brucellosis is a zoonosis caused by gram-negative bacteria of the genus *Brucella*. Its incidence in France has declined to fewer than 50 cases per year, or 0.65 per million inhabitants [1]. However, it remains prevalent at European borders, particularly in the Mediterranean basin and Middle East. Of the 587 reported cases in France between 2002 and 2023, 213 (87.4 %) were linked to travel to endemic countries or consumption of contaminated products. About 10 % involved children, fewer than half under nine [1]. In France, the remaining cases are primarily among laboratory staff [2], [3]. Transmission is primarily foodborne through the ingestion of infected raw or unpasteurized dairy products and meat. Human-to-human transmissions are very rare and include mother-to-fetal transmission through placental circulation or breastfeeding, and mothers may not be symptomatic [4], [5], [6]*.* Brucellosis is a rare cause of fetal loss, spontaneous prematurity, and early or late onset sepsis, with or without chorioamnionitis [[5], [6], [8], [9], [10]]. The large number of infected organs leads to diverse clinical presentations. About 40 neonatal cases have been reported worldwide since 1941, the last in France in 1990 [10]. This article presents a literature review and a case managed in Nantes University Hospital.

## Presentation of the case and literature review

### Methods

The case was reported per CARE guidelines. The study followed CNIL MR004 regulations and received ethics approval. Oral parental consent was obtained, and they received a detailed written information note. A PubMed search was conducted using "congenital brucellosis" OR "neonatal brucellosis" (2000 to June 2024). Of 97 articles, 13 met inclusion criteria (neonates under 3 months, maternal-fetal transmission, post-2000, with full text available).

### The case

A 37-year-old woman was hospitalized in October 2023 to the Nantes University Hospital for premature rupture of membranes (PROM) at 22 weeks of gestation (WG), without fever. Prior to admission, she had received cefixime (200 mg twice daily for 7 days) for asymptomatic bacteriuria due to *Klebsiella pneumoniae* (susceptible to cephalosporins) on October 6th, with no complications. On admission to the high-risk pregnancy unit for PROM, vaginal culture was negative for *Streptococcus agalactiae* but positive for *Escherichia coli* (susceptible to cephalosporins and gentamicin) and *Enterococcus faecalis* (intrinsically resistant to cephalosporins, susceptible to amoxicillin), which was not specifically taken into consideration. She remained clinically stable, with clear amniotic fluid, no fever, no signs of infection, no uterine contractions, and active fetal movements. Two courses of antenatal corticosteroids were administered for fetal lung maturation. Pre-term laboratory evaluation showed an inflammatory syndrome, but no blood cultures were obtained in the absence of fever or sepsis.

Labor started spontaneously at 26 WG + 3 days, again without maternal fever. A female neonate (895 g, 77th percentile) was born afebrile. The early postnatal course was consistent with extreme prematurity. The early postnatal respiratory distress, due to severe hyaline membrane disease, requiring two surfactant instillations in the delivery high oxygen requirements. Empiric antibiotic therapy with Cefotaxime (100 mg/day) and Gentamicin (5 mg/kg/day for 2 days) was initiated in the newborn at birth due to unexplained pre-term delivery.

In this context, two maternal blood cultures and placenta blood culture were performed postpartum. Laboratory testing revealed WBC 19 × 109 /L, CRP 22 mg/L, platelets 353 × 109 /L, and inflammatory anemia (Hb 10.6 g/dL, MCV 87 fl), liver function tests and coagulation were normal, with no hemolysis. The mother developed fever a few hours postpartum and received Amoxicillin (2 g three times daily) and Gentamicin (5 mg/kg/day) after specimen collection.

Meanwhile, the neonate’s clinical condition remained unchanged: ventilation was stable under CPAP, hemodynamics were stable, she remained afebrile, and enteral feeding was well tolerated with no abdominal abnormalities. Her blood culture at birth was negative, inflammatory biomarkers were within normal limits, and neonate’s empiric antibiotics were discontinued after 48 h.

Two maternal blood cultures after delivery became positive for *Brucella melitensis* at 46 and 68 h, as the placental culture at 48 h. Each sample was incubated in a glucose-enriched solid medium (chocolate agar with growth factors) under anaerobic conditions. After culture, direct Gram staining was performed, followed by pathogen identification using mass spectrometry. Brucella is an intracellular pathogen resistant to cephalosporins, but susceptible to rifampicin, doxycycline, and trimethoprim-sulfamethoxazole. The mother’s antibiotic regimen was modified on day 4 in accordance with current guidelines for adult patients [11] i.e., doxycycline (100 mg twice daily) and rifampicin (600 mg twice daily) for six weeks, combined with gentamicin (5 mg/kg/day) for the first seven days. The mother became afebrile, muscle aches resolved, and no complications occurred. Breastfeeding was contraindicated from the first positive maternal blood culture, although pasteurized milk was provided and *Brucella* PCR was negative. The neonate had a favorable course with gradual reduction of ventilatory requirements, and she regained her birth weight on day 9 of life.

On 13 days of life, the infant developed a fever, abdominal distension, apnea and desaturation despite daily caffeine therapy. Chest X-ray revealed persistent features of hyaline membrane disease without new findings. Laboratory results showed leukocytosis (20 G/L, 48 % lymphocytes), negative CRP, and cerebrospinal fluid parameters within normal limits for age (21 cells, glycorrhachia/glycemia ratio 0,4, protein 1,07 g/L), with negative CSF culture and PCR. Blood cultures obtained on the 13th, 14th, and 15th day were positive for *B. melitensis* within 46–78 h. Repeated blood cultures remained sterile from day 16th.

Antimicrobial management of this congenital brucellosis case was guided by the National Resource Center for the Brucella (NRC) [11]. As doxycycline and TMP/SMX are considered unsafe for prolonged neonatal use, she was initially treated with intravenous initial combination of rifampicin (20 mg/kg/day twice a day) and cefotaxime (200 mg/kg/day in three doses), followed by the oral rifampicin combined with TMP/SMX (8/40 mg/kg/day twice a day) starting at four weeks of life (*i.e.* 31 weeks corrected age) under close laboratory monitoring. As blood cultures rapidly established the diagnosis, serological testing was not performed. Breastfeeding was not resumed despite negative prolonged cultures (15 days) and PCR of breast milk, in accordance with maternal preference and the need for maternal doxycycline therapy.

No secondary localizations were identified: joint examination was unremarkable, and repeated cardiac, abdominal, and transfontanellar ultrasounds were normal. Brain MRI at 36 weeks corrected age was also normal, allowing discontinuation of treatment after six weeks of antibiotics from the first negative blood culture. The case was reported to the health authorities as required.

Maternal history indicated residence in France, origin from Western Sahara, and raw milk cheese during a trip to Spain in the first trimester.

The neonate progressed well under antibiotic therapy. No recurrence of fever after the initial peak. The hospital stay was complicated by bronchopulmonary dysplasia requiring prolonged non-invasive ventilation and supplemental oxygen at discharge in the context of extreme prematurity. Neurodevelopment was appropriate for corrected age of seven months. She demonstrated appropriate growth along the 50th percentile.

### Literature review

Maternal exposure histories—mostly involving unpasteurized dairy or animal contact— varied symptoms, from asymptomatic cases to fever, malaise, or complications like premature rupture of membranes (Table 1).

**Table 1:** Literature review of cases of congenital infections – Mother’s histories.

***References***	**Country**	**Date**	**Species**	**Culture / PCR**	**Incubation**	**Agglutination titer**	**Age at diagnosis**	**Contamination**	**Maternal symptoms and treatment**
*Mesner**[7]*	Israel	2007	*B.melitensis*	M+ **N +**	4 days	**M+ : 1/20 1/60** **N-**	**4**^**th**^ day	1st trimester: Sheep and goat breeding, consumption of unpasteurized dairy products	**11 WG:** fever, hepatomegaly, hepatitis (negative viral serologies). **22 WG:** fever, chronique hepatitis → Amoxicilline - Acide clavulanique **24 WG:** hemorrhage preterm delivery due to placenta previa → total hysterectomy. Shock, ARDS, adrenal insufficiency, jaundice.
*Ceylan**[9]*	Turkey	2011	NR	**M+** 1 mo before delivery **M-** postnatal **N-**	NR	**M+ : 1/1280** (after delivery), **1/640** (milk) **N + :** 1/160	**24 WG**, 1 mo before delivery	10 WG: Consumption of infected sheep meat	**18 WG:** Anorexia, malaise, vomiting, vaginal bleeding **24 WG:** Brucellosis diagnosis → Rifampicine + TMP-SMX **28 WG:** spontaneous pre-term delivery under treatment **3rd wk post-delivery:** Relapsing Brucellosis → 2nd Rifampicine+ TMP-SMX
*Glocwicz**[4]*	USA (Texas)	2010	*B.melitensis*	**M-** **N +**	NR	**M+ :** 1/640 (pp) **N:** NR	**39**^**th**^ day pp	1st trimester: Travel to Bosnia-Herzegovina during pregnancy	No symptoms during pregnancy **24 WG:** spontaneous pre-term delivery **39**^**th**^**day pp:** Brucellosis diagnosis → Rifampicine+ TMP-SMX 6 wks
*Dogan**[16]*	Turkey	2010	NR	**M+** at delivery **N +** at delivery	10 days	**M+ :** 1/1280 **N + :** 1/640	**10**^**th**^ day pp	Consumption of unpasteurized cheese	**Before delivery:** Arthralgia for a month **31 WG:** spontaneous preterm delivery **10**^**th**^**day pp:** brucellosis diagnosis → Rifampicine+ TMP-SMX 6 wks
*Mosayebi**[5]*	India	2005	*B. abortus* (TMP-SMX Resistance)	**M+** at delivery **M-** 1 wk pp **N +**	NR	**M+ :** 1/160 **N +:** 1/320	**26 WG:** delivery	Cow and goat breeding, and consumption of unpasteurized dairy products	No symptoms during pregnancy **26 WG**: spontaneous pre-term delivery Diagnosis at delivery → Rifampicin + Doxycycline 6 wks
*Sarafidis**[6]*	Greece	2007	*B.melitensis*	**M-** at delivery **N +**at delivery	6 days	**M+ :** 1/160	**6**^**th**^ day pp	Sheep and goat breeding	No symptoms during pregnancy **37 WG**: spontaneous preterm delivery with amniotic fluid disorder
*Alsaif**[8]*	Saudi Arabia	2018	*Brucella* sp.	**M-** **N +**	3 days	**M+** **N-**	**3rd** day pp	1st trimester: ingestion of raw camel milk and aunt had brucellosis	During pregnancy: hip pain without fever **26 WG**: spontaneous preterm delivery
*Koklu**[17]*	Turkey	2006	*B.melitensis*	**M+** at delivery **N +a**t delivery	5 days	**M+ :** 1/320 **N +:** 1/320	**5**^**th**^ day pp	Cows/goats at home mother: unpasteurized dairy father: brucellosis 2 yrs prior	No symptoms during pregnancy but no antenatal care **18 WG:** undefined premature labor, rupture of membranes, vaginal bleeding **22 WG:** spontaneous preterm delivery
*Cacace**[18]*	Argentina	2013	*B.melitensis*	**M:** NR **N +**	5 days	**M+ :** 1/400 **N-:** 1/50	**1 mo 22 days:** Due to initial misidentification	Breeder family (cows/goats/pigs) mother: unpasteurized dairy	No symptoms during pregnancy and no antenatal care **40 WG:** complicated vaginal delivery Breastfeeding up to diagnosis
*Mohzari**[19]*	Saudi Arabia	2020	*B.melitensis* and *B.abortus*	**M:** NR **N +:**at delivery	6 days	**M:** NR **N +:** 1/160	**6**^**th**^ day pp	1st trimester: unpasteurized sheep's milk breeder family with history of brucellosis	**21 WG:** Headache, anorexia, malaise, sweating, abdominal pain **24 WG**: Urinary infection, antibiotics (unspecified) **27 WG + 6d**: PRM **29 WG:** spontaneous pre-term delivery and **6**^**th**^ day pp → treatment
*Xu**[20]*	China	2021	*B.melitensis*	**M+ :** at delivery PCR+ : placenta **N +:**at 26th day (blood and CSF)	4 days	**M+ :** at delivery **N +**at 26th day	**4**^**th**^ day pp (mother) **26**^**th**^ day pp (neonate)	Farm life with sheep without close contact during pregnancy. Family and animals’ illness.	During pregnancy: asthenia, arthralgia for 2 wks **37 WG + 4d:** delivery by cesarean for chorioamnionitis (fever) Breastfeeding for four days **4**^**th**^**day pp:** Brucellosis diagnosis → Rifampicin + Doxycycline 6 wks
*Al-Faifi**[21]*	Saudi Arabia	2022	NR	**M+** **N +:** at delivery	5 days	**N-:** 1/80 at delivery	Before delivery	Not described	During pregnancy: Brucellosis diagnosis and treatment (unspecified) **30 WG:** spontaneous pre-term delivery No breastfeeding
*Zhao**[12]*	China	2019	*B.melitensis*	N +at delivery PCR+ in blood	3 days	**M+** **N-**	**3rd** day pp	Parents rode camels 12 mo before pregnancy; father: orchitis + fever.	**30 WG:** weakness in lower extremities and hip joint pain **34 WG:** spontaneous preterm delivery

M: mother; N: neonate; months: month; “+ ”: positive; “-“: negative; WG. weeks of gestation. SAT, seroagglutination titer; PRM, Premature rupture of membrane; ARDS: acute respiratory distress syndrome; TMP-SMX: trimethoprim/sulfamethoxazole, pp: postpartum; NR: not reported; CSF: cerebrospinal fluid; PCR: polymerase chain reaction; wk: weeks, d: day

Most diagnoses are made at birth and *B. melitensis* as the predominant species. Newborns frequently showed respiratory distress, sepsis, hepatosplenomegaly, and in some cases neurological complications, with a wide range of gestational ages and birth weights reported (Table 2).

**Table 2:** Literature review of cases of congenital infections – Infants histories.

***References***	**Sex**	**Term (WG)**	**Birth weight (g)**	**APGAR 1′/5′**	**Newborn symptoms**	**Treatment**	**Evolution**
*Mesner*	NR	24	NR	2 / NR	**At birth:** ARD, emphysema, PAH, septic shock, PDA	Rifampicin + Gentamicin + Ampicillin	Death at 20 days (candidemia)
*Ceylan*	NR	28	970	NR	**At birth:** lethargic and dyspneic	Rifampicin + TMP/SMX 6 weeks	No sequelae
*Glocwicz*	M	24	480	NR	**At birth**: sepsis	**Empiric at birth:** Ampicillin + gentamicin 10 days (efficacity unspecified) **Day 39:** Rifampicin + TMP/SMX 8 weeks	No sequelae at age 3
*Dogan*	F	31	1480	6 / 8	** h pp:** Intubated ARD, HSMG, Biological Inflammation	Rifampicin + TMP/SMX 6 weeks (3 PO)	No sequelae at age 1
*Mosayebi*	M	26	900	4 / 7	**At birth:** sepsis and ARD	Rifampicin (10 mg/kg/j) + Imipenem (20 mg/kg/j)	Death at 21 days (PNO)
*Sarafidis*	F	37.5	3260	NR	**At birth:** sepsis, ARD, alveolar-interstitial syndrome, cutaneous rash and HSMG	Rifampicin (15 mg/kg/d) + TMP-SMX (10/50 mg/kg/d) 21 days + Gentamicin for the first 6 days	No sequelae at age 1
*Alsaif*	M	26	845	NR	**At birth:** Neonatal sepsis and ARD	Rifampicin + TMP-SMX 6 weeks PO + Gentamicin first 2 weeks	No sequelae at 19 months of age
*Koklu*	F	32	1100	4 / 9	**At birth:** HBP, HSMG, early jaundice, hepatitis, neonatal alveolar-interstitial syndrome	TMP-SMX + Gentamicin IV 14 days, then TMP-SMX +Rifampicin IV 6 days, then TMP-SMX PO 35 days	No sequelae at 13 months of age
*Cacace*	F	40	2750	6 / 7	**At birth:** sepsis, ARD, hypothermia, cyanosis, hypoxic ischemic encephalopathy, seizures, HSMG	TMP-SMX (10 mg/kg/d) + Rifampicin (20 mg/kg/d) 6 weeks	No sequelae at 13 months of age
*Mohzari*	F	29	1085	6 / 8	**At birth**: Sepsis and intubated ARD	Rifampicin (10 mg/kg/d) + Ciprofloxacin (10 mg/kg/d) × 6 wks IV + Gentamicin × 2 weeks Day 17: Respiratory deterioration → Meropenem × 1 wk	
*Xu*	F	37	3300	NR	Unrecognized maternal infection at birth **Day 34**: Brucella meningitis **1**^**st**^**relapse (Day 70)**: fever, +blood culture, SAT > 1:400 **2**^**nd**^**relapse (Day 120)**: fever, +blood culture **3**^**rd**^**relapse:** During sequential therapy; fever, hepatitis; chronic course without secondary localization	**1**^**st**^**treatment:** Meropenem (2w) + Rifampicin (6wks) **→ 1st relapse**: Rifampicin + TMP-SMX (8wks) **→ 2nd relapse**: 3 cycles Rifampicin + TMP-SMX (6 wks each) **→ Chronic infection:** 3 courses Rifampicin + TMP-SMX (6 Wks each, 1 wk interval)	No sequelae
*Al-Faifi*	F	< 36	NR	NR	**At birth:** Severe ARD	Rifampicin + Gentamicin + Ciprofloxacin	No sequelae
*Zhao*	M	35	2000	9	** h pp**: needs oxygen and had poor response to stimuli, HSMG, hepatitis, hyperleukocytosis CSF: cellular reaction without bacteria	TMP-SMX po (25 mg/kg/d) + Rifampicin po (10 mg/kg/d) for 6 weeks + beta-lactam IV 4 weeks	No sequelae at 2 months follow-up

WG. weeks of gestation. SAT, seroagglutination titer; PRM, Premature rupture of membrane; NR, not reported; ARD, Acute respiratory distress; PAH, Pulmonary arterial hypertension; PDA, Persistent ductus arteriosus; HSMG, Hepatosplenomegaly; HBP, High blood pressure; TMP-SMX, trimethoprim/sulfamethoxazole. CFS: cerebrospinal fluid, pp: postpartum; IV: intravenous; PO: per os; wk: week; wks: weeks.

In the reported cases (Table 3), blood cultures were positive between 3 and 10 days of incubation. In seven cases, a seroagglutination test was performed, which came back negative in three premature infants with delayed positive blood cultures [7], [8], [12]. Negative serology should not preclude diagnosis, especially in preterm infants who may not have developed their own immune response or received transplacental antibodies [8]. Although serological testing is increasingly considered outdated in France due to its limited sensitivity and specificity compared to molecular and culture-based techniques, it remains a cornerstone of diagnosis in low-resource settings. In such contexts, the combined use of multiple immunological assays is recommended to improve diagnostic accuracy [13]. The sensitivity of blood cultures varies according to the clinical stage: it is over 80 % in the acute phase of the disease [11]. Molecular biology is not widely reported, due to its recent development and varying availability in different countries. However, it is now the gold standard in France when blood cultures fail, or antibiotics have been started. It can be performed on any type of sample, but its sensitivity and specificity are far better on blood cultures. Species identification may be performed with multiplex PCR used by the National Resource Center [3].

Most neonatal cases in the literature were treated with rifampicin and TMP/SMX from the outset without adverse effects such as agranulocytosis or nuclear jaundice (Table 3). This combination has been standard since 1993 [14]. If we exclude the article of Xu et al., which refers to chronic disease, the median duration of antibiotic treatment is six weeks.

Apart from congenital cases, three cases of infant brucellosis linked to breastfeeding and one case linked to blood transfusion have been reported since 2000. In France, a case of food transmission in a 16-month-old infant was reported in 2015 [15]

## Discussion

A review of the literature identified approximately 40 reported cases of congenital or neonatal brucellosis worldwide since 1941, with diverse clinical presentations ranging from early sepsis to late-onset manifestations. The majority were linked to *Brucella melitensis.*

Factors promoting the spread of the disease in certain regions include the lack of access to milk pasteurization, proximity to animals in agricultural areas, and difficulties in applying hygienic and dietary measures, especially for pregnant women. Additionally, in some parts of the world, the belief in the benefits of raw milk (specifically raw camel milk) remains deeply rooted in traditional culture [8].

In this case, clinical recognition of infection was delayed until day 13 of life, despite maternal cultures being positive at delivery. This underscores how congenital brucellosis may present late with non-specific neonatal symptoms and highlights the importance of maintaining early vigilance and considering blood cultures when maternal risk factors are present.

The newborn’s initial good clinical course may have been related to bi-antibiotic treatment, with secondary deterioration upon discontinuation. The symptomatic manifestation 13 days after birth is unusual for a congenital infection and could suggest secondary contamination by breastfeeding, which was not the case here. Documenting bacteremia in a premature newborn is challenging due to often insufficient blood culture volumes. Given the non-specific symptoms of brucellosis, empiric antibiotic therapy may be justified, especially in confirmed chorioamniotitis with *B. melitensis.*

Regarding aminoglycosides, a combination of gentamicin and TMP/SMX has been successfully used in neonatal cases since the 1990s (26). The NRC emphasizes the value of using triple therapy with an aminoglycoside, especially if there are secondary localizations (2), though not indicated here.

Tube enteral feeding can decrease absorption of rifampicin. We monitored plasma concentration, but it remained low (less than 8 μg/mL) despite increasing the dose to 20 mg/kg/day. Given the good clinical evolution, the dosage was not modified.

Continued breastfeeding may be safe if both mother and child are treated [8], but in these 13 cases, there were no reported cases of breastfeeding after a congenital infection diagnosis.

## Conclusion

This rare French case of first-trimester transplacental transmission highlights the importance of considering brucellosis in neonatal sepsis or maternal fever. Maternal travel and dietary history must be assessed. If maternal blood culture is positive, the newborn should be thoroughly screened and possibly treated empirically.

## Ethical approval

Ethical approval was obtained by the Nantes Health Ethics Group (GNEDS) on 11/14/24.

## Sources of funding

No funding

## Financial support

None

## Consent

oral consent, anonymous case report

## Informed Consent

Verbal informed consent was obtained from mother’s minor patient for anonymized information to be published in this article. The family gave his oral consent, and they received a detailed written information note, following which they expressed no objection.

## Animal Welfare

This research did not involve the use of animals.

## Funding

This research received no specific grant from any funding agency in the public, commercial, or not-for-profit sectors.

## Ethical Statement

Ethical approval for this case was obtained from Nantes Health Ethics Group (Groupe Nantais d’Ethique dans le Domaine de la Santé - GNEDS) Number 24–132–11–141 / E. Launay, L. Ozcan.

## CRediT authorship contribution statement

**Cyril Flamant:** Writing – review & editing, Validation. **Jean-Philippe Lavigne:** Writing – review & editing, Validation, Supervision, Resources. **Stéphane Corvec:** Writing – review & editing, Validation, Resources. **Valentin Pineau:** Writing – review & editing, Validation, Resources. **ozcan lisa:** Writing – original draft, Resources, Project administration, Methodology, Investigation, Formal analysis, Data curation, Conceptualization. **David Malorey:** Writing – review & editing, Validation, Supervision. **Elise Launay:** Writing – review & editing, Validation, Supervision, Project administration, Methodology. **Constance Bridonneau:** Visualization, Validation, Investigation. **Perceval Lise:** Writing – review & editing, Validation. **Mona Leroux:** Visualization, Validation, Investigation.

## Declaration of Competing Interest

The authors declare that they have no known competing financial interests or personal relationships that could have appeared to influence the work reported in this paper.
